# Circulating levels of adiponectin and extent of coronary artery disease in patients undergoing elective coronary angiography

**DOI:** 10.1590/1414-431X20176738

**Published:** 2017-11-30

**Authors:** R.A. Souza, C.M.R. Alves, C.S.V. de Oliveira, A.F. Reis, A.C. Carvalho

**Affiliations:** 1 Cardiologia Intervencionista, Universidade Federal de São Paulo, São Paulo, SP, Brasil; 2 Disciplina de Endocrinologia, Universidade Federal de São Paulo, São Paulo, SP, Brasil; 3 Disciplina de Cardiologia, Universidade Federal de São Paulo, São Paulo, SP, Brasil

**Keywords:** Adipose tissue, Adipokines, Adiponectin, Coronary artery disease, Atherosclerosis

## Abstract

Adiponectin (APN), an adipose tissue-released adipokine with demonstrated anti-inflammatory and anti-atherogenic properties, is encoded by a gene whose polymorphisms are associated with presence of coronary artery disease (CAD). Serum APN levels are inversely related with presence and complexity of CAD. Within this context, we sought to compare levels of total APN and its high molecular weight form (HMW APN) according to clinical presentation and extent of CAD in patients undergoing elective cardiac catheterization. From March 2008 to June 2010, clinical data and blood samples for APN and HMW APN measurements were collected from 415 subjects undergoing cardiac catheterization at two tertiary centers. CAD extent was estimated by the number of coronary arteries with significant stenosis (≥70% obstruction in a major coronary artery) and by Duke Jeopardy Score (DJS). Serum APN levels were similar between groups with stable or unstable CAD (APN 9.20±5.88 *vs* 9.47±6.23 μg/mL, P=0.738, and HMW APN 5.31±3.72 *vs* 5.91±4.16 μg/mL, P=0.255), even after stratification by the number of arteries involved (single-vessel *vs* multivessel disease: APN 9.39±5.76 *vs* 9.26±6.27 μg/mL, P=0.871; HMW APN 5.29±3.79 *vs* 5.83±4.04 μg/mL, P=0.306) and DJS score (APN, P=0.718; HMW APN, P=0.276). We conclude that APN and HMW APN serum levels are similar across clinical presentations and different extents of CAD, despite being significantly lower in the presence of obstructive CAD.

## Introduction

In the spectrum of cardiovascular disease, coronary artery disease (CAD) plays a predominant role, accounting for one third to half of all cases, constituting a serious public health problem and one of the leading causes of mortality worldwide (1–3).

Among the major risk factors for CAD, the prevalence of obesity has increased markedly in recent years, to the extent that this condition is now considered a worldwide epidemic; as of 2014, obesity was estimated to affect 600 million people globally (4). Adipose tissue, rather than serving merely as an energy store, has an essential role in regulating a wide range of metabolic functions through secretion of biological mediators known as adipocytokines (or adipokines). Among these, adiponectin (APN) is the most abundant adipocytokine in plasma. Unlike concentrations of other adipocytokines, serum levels of APN are paradoxically reduced in the presence of obesity, diabetes, and CAD (5).

APN appears to modulate the interaction between classic risk factors and atherosclerosis (6). Studies in several diverse populations have demonstrated a correlation between lower APN levels and increased prevalence and extent of CAD (7–11), as well as increased risk of acute myocardial infarction (AMI) in men over long-term follow-up (12). High molecular weight APN (HMW APN), which is the major active form of this protein, seems to be selectively reduced in the presence of CAD (13), besides being implicated with CAD extent and the risk of future cardiovascular events (10).

Thus, APN and/or HMW APN might be useful as early biomarkers of cardiovascular risk in general and atherosclerosis in particular, as well as a possible predictor of adverse cardiovascular events in patients with CAD.

In Brazilian populations, lower APN levels have been associated with increased prevalence of CAD (14) and risk of major cardiovascular events - such as nonfatal AMI, nonfatal stroke, and death - within 1 year of acute coronary syndrome (ACS) (15). However, to date, the potential association of APN levels with clinical presentation and extent of CAD have not been evaluated in this diverse, multiracial population.

Within this context, the aim of this study was to compare serum levels of APN and HMW APN form according to clinical presentation and extent of obstructive CAD.

## Material and Methods

### Study population

From March 2008 to June 2010, 415 patients undergoing elective cardiac catheterization at two tertiary centers were included in the study. Patients with chronic kidney disease (creatinine clearance <60 mL/min), active inflammation, malignancy, or a history of coronary artery bypass grafting or thyroid disease were excluded. Clinical data were collected on the day of the procedure. Hypertension, diabetes mellitus, metabolic syndrome, and dyslipidemia were defined as per the Brazilian Society of Cardiology and American Diabetes Association. After written informed consent had been obtained, blood samples were drawn for APN and HMW APN measurement, as described previously (14). Patients were divided into two groups according to the presence or absence of obstructive CAD. The obstructive CAD group was subdivided by clinical presentation: stable (defined as the presence of stable angina and/or documented silent ischemia) or unstable (defined as the presence of acute coronary syndrome with or without ST segment elevation >24 h after onset). The study protocol was approved by the local Ethics Committee (CAAE 03056812.5.0000.5505, approval #180.635) and followed the provisions of the Declaration of Helsinki. As the present study simply reviewed existing data, the Ethics Committee waived the requirement of obtaining informed consent again from the participants of the parent study.

### Laboratory tests

Blood samples were collected before cardiac catheterization and after an overnight fast. HMW APN and APN levels were measured with commercially available ELISA test kits (EZHMWA-64k and EZHADP-61K respectively; Millipore, USA). The intra- and inter-assay coefficients of variation were 3.41 and 9%, respectively (sensitivity 0.5 ng/mL) for HMW APN and 7.4 and 10.6%, respectively (sensitivity 0.78 ng/mL) for APN.

### Analysis of cine coronary angiograms

Analysis was performed by an individual review of each angiogram by the first author and comparison with the official angiogram report. In case of disagreement, final review was performed by a third examiner. Obstructive CAD was defined as the presence of at least one stenotic lesion involving ≥70% of the reference diameter of the vessel (the percentage at which significant hemodynamic abnormalities begin to occur), by visual estimation. The other lesions were classified as non-obstructive CAD.

The extent of CAD was quantified by classification as single-vessel or multivessel disease, as well as by the Duke Jeopardy Score (16). In brief, the coronary circulation was divided into eight segments: left main coronary artery (LMCA), proximal third of the left anterior descending artery (LAD), middle/distal third of the LAD, first major diagonal branch (DG1), first major septal perforator, proximal third of the circumflex artery (LCx), first major circumflex marginal branch (MG1), and right coronary artery (RCA). Each distal segment with an obstructive lesion was assigned a score of 2. The final score was defined by the sum of scores in each segment, and thus ranged from 2 to 12 points for patients with at least one obstructive lesion. In case of significant stenosis in a proximal segment, all distal segments were considered affected; e.g., proximal-third LAD stenosis, a score of 6 points (corresponding to the distal segments) was assigned. For purposes of statistical analysis, scores were classified into three categories: low (2 and 4), intermediate (6 and 8), and high (10 and 12).

### Statistical analysis

Data were recorded in Microsoft Excel 2010¯ spreadsheets (Microsoft Corp., USA) for subsequent analysis. Distributions of continuous variables were reported as means±SD or median (interquartile range). The Student's *t-*test or Mann-Whitney *U* test and ANOVA were used for between-group comparisons as appropriate, depending on the normality of data distribution. Categorical variables were expressed as absolute and relative frequencies, and the chi-square test was used for comparisons. Analyses were performed in the IBM¯ SPSS Statistics 20.0 software environment (IBM Corp., USA), with a significance level of α <0.05 and statistical power of 80%.

## Results

The general characteristics of the 415 patients included in the study are shown in Table 1. Overall, the sample was predominantly male (57.3%) and white (60%), with a mean age of 58.5±9.92 years, and a large proportion of patients with diabetes (43.4%). Of the 415 patients included, 224 (54%) had at least one obstructive lesion on angiographic assessment; of these, 121 (54%) had stable disease.

**Table 1.** Demographic, clinical, and laboratory characteristics of patients undergoing elective coronary angiography, according to the presence of coronary artery disease (CAD).

Variable	Obstructive CAD (n=224)	Non-obstructive CAD (n=191)	P
Male gender, n (%)	140 (62.5%)	98 (51.3%)	0.022
Age (years)	59.2±9.86	57.6±9.95	0.085
BMI (kg/m^2^)	27.7±4.51	28.2±5.22	0.265
Obese (BMI ≥30 kg/m^2^)	67 (29.9%)	66 (34.6%)	0.510
Race, n (%)			0.346
White	138 (61.6%)	111 (58.1%)	
Brown	25 (11.2%)	33 (17.3%)	
Black	59 (26.3%)	45 (23.6%)	
Asian	2 (0.9%)	2 (1.0%)	
Past medical history, n (%)			
Diabetes mellitus	99 (44.2%)	81 (42.4%)	0.714
Impaired fasting glucose	93 (41.5%)	62 (32.5%)	0.057
Hypertension	184 (82.1%)	155 (81.2%)	0.795
Hypertension	205 (91.5%)	160 (83.8%)	0.016
Metabolics	97 (43.3%)	65 (34%)	0.054
Family history of CAD	89 (39.7%)	56 (29.3%)	0.027
Smoking	84 (37.5%)	77 (40.3%)	0.558
Sedentary lifestyle	172 (76.8%)	125 (65.4%)	0.011
Past AMI	96 (42.9%)	42 (22%)	<0.001
Past stroke	8 (3.6%)	6 (3.1%)	0.809
Clinical presentation, n (%)			
Acute coronary syndrome	103 (46%)	79 (41.4%)	0.344
Laboratory findings			
Fasting glucose (mg/dL)	123.6±48.9	114.4±36.3	0.029
HbA1c (%)	6.6±2.0	6.4±1.3	0.201
Triglycerides (mg/dL)	164.9±93.3	152.6±91.8	0.180
HDL cholesterol (mg/dL)	37.0±10.4	39.4±11.4	0.028
LDL cholesterol (mg/dL)	103.5±35.3	101.5±35.2	0.565
CrCl (mL·min^-1^/1.73m^2^)	93.7±24.0	101.5±35.2	0.009
HMW APN (μg/mL)	5.59±3.93	7.08±6.1	0.003
APN (μg/mL)	9.32±6.03	11.34±8.6	0.004

Data are reported as means±SD. BMI: body mass index; AMI: acute myocardial infarction; HDL: high density lipoprotein; LDL; low density lipoprotein; CrCl: creatinine clearance; HMW APN: high molecular weight adiponectine; APN: adiponectine (*t-*test, Mann-Whitney *U* test, and chi-square test, as appropriate).

Regarding clinical presentation, the stable and unstable CAD groups were similar in distribution of clinical and demographic characteristics. Angiographic findings and Duke Jeopardy Score distributions are shown in Table 2. There was no statistically significant difference in levels of APN (stable, 9.20±5.88 μg/mL; unstable, 9.47±6.23 μg/mL; P=0.738) or HMW APN (stable, 5.31±3.72 μg/mL; unstable, 5.91±4.16 μg/mL; P=0.255) between the two groups.

**Table 2.** Angiographic findings and Duke Jeopardy Score of patients undergoing elective coronary angiogram according to clinical presentation of obstructive coronary artery disease.

Variable	Stable (n=121)	Unstable (n=103)	P
Number of affected vessels			0.768
Single-vessel	54 (44.6%)	48 (46.6%)	
Multi-vessel	67 (55.4%)	55 (53.4%)	
Duke Jeopardy Score			0.747
Low	73 (60.3%)	65 (63.1%)	
Intermediate	31 (25.6%)	27 (26.2%)	
High	17 (14%)	11 (10.7%)	

Statistical analysis was done with the chi-square test.

**Figure 1.**
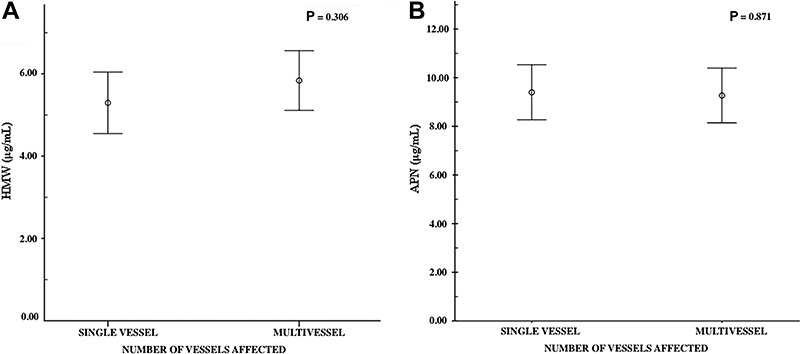
Association of high molecular weight (HMW) adiponectin (APN) (*A*) and APN (*B*) levels with number of affected coronary vessels. Data are reported as means±SD (Mann-Whitney *U* test)

**Figure 2.**
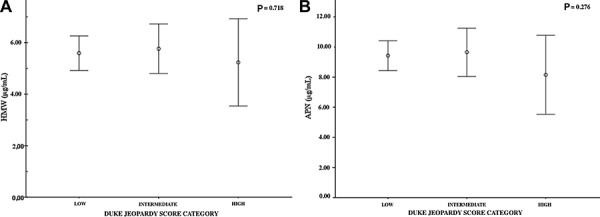
Association of high molecular weight (HMW) adiponectin (APN) (*A*) and APN (*B*) levels with Duke Jeopardy Score. Data are reported as means±SD (ANOVA).

Comparison between serum levels of these biomarkers and extent of obstructive CAD, as estimated by the number of affected vessels (single-vessel *vs* multivessel, Figure 1A and B), did not reveal differences in APN (9.39±5.76 μg/mL *vs* 9.26±6.27 μg/mL; P=0.871) or HMW APN (5.29±3.79 *vs* 5.83±4.04 μg/mL; P=0.306). Likewise, stratification by Duke Jeopardy Score into low, moderate, and high groups (Figure 2A and B) did not reveal any significant difference with levels of APN (9.42±5.87 *vs* 9.65±6.09 *vs* 8.15±6.76 μg/mL, P=0.718) or HMW APN (5.59±3.98 *vs* 5.76±3.66 *vs* 5.23±4.36 μg/mL, P=0.276).

Nevertheless, there were significant differences between the obstructive and non-obstructive CAD groups in levels of APN (9.32±6.03 *vs* 11.34±8.6 μg/mL, P=0.004) and HMW APN (5.59±3.93 *vs* 7.08±6.1 μg/mL, P=0.003).

## Discussion

The findings of this study do not demonstrate any statistically significant difference between low serum levels of APN or HMW APN regarding clinical presentation or angiographic extent of CAD. However, by using ≥70% stenosis as a cutoff for definition of obstructive CAD, the findings of this study provide further evidence of the relationship between low APN levels and prevalence of obstructive CAD in a group of patients with high cardiovascular risk.

The use of biomarkers to diagnose or estimate the extent and severity of CAD is both academically and clinically interesting. APN is a particularly promising such biomarker, as it is known to act on the interaction between atherosclerosis and classic CAD risk factors, has a demonstrable relationship with increased prevalence and extent of CAD, and has been associated with increased risk of AMI in different populations (7–12). However, the literature is still conflicting, and there is no established threshold for hypoadiponectinemia, which hinders comparison between different studies. In addition, studies of the relationship between APN and CAD in the Brazilian population are scarce, and, despite corroborating this association, have failed to address other important aspects, such as clinical presentation and disease extent (14,15).

The present study is an extension of a previous analysis by Oliveira et al. (14), which found an association of reduced APN levels with certain genetic polymorphisms and presence of CAD, regardless of blood glucose levels and clinical presentation. However, this analysis defined CAD as the presence of any visible atherosclerotic lesion on coronary angiography, regardless of the percentage of luminal stenosis or number of vessels affected. In the present study, a different set of criteria was adopted for the same population. An additional prospective analysis of a Brazilian population (15) found that lower APN levels were associated with new-onset cardiovascular events within 1 year only in patients with acute coronary syndrome. This highlights the role of APN as a predictor of cardiovascular events. However, these analyses did not take into account the anatomic severity or extent of CAD.

Our findings corroborate previous reports suggesting that risk of obstructive CAD is increased in individuals with low APN levels (17,18). Further evidence indicates that low levels of this adipokine may be associated with a two-fold prevalence of angiographically visible CAD, regardless of other cardiovascular risk factors, including diabetes mellitus, dyslipidemia, hypertension, smoking, and high BMI (7).

Nevertheless, no consensus has been reached as to this association. It has been called into question by Sattar et al. (8 [Bibr B09]) in a prospective study and meta-analysis, which revealed lower odds of CAD in patients with lower APN levels (OR=0.89, 95%CI=0.67–1.18). The same group of researchers assessed the relationship between incidence of CAD in women enrolled in the British Women's Heart and Health Study and lower HMW APN and APN levels, and found no significant association (19). More recently, Amirzadegan et al. (20) also found no association between APN levels and presence of CAD.

Clearly, the data available are still conflicting, and additional, robust studies are needed to elucidate this relationship. However, we believe that APN may become a useful biomarker that can provide relevant information to daily clinical practice, aiding in identification of cases with higher odds of obstructive CAD, particularly in patients in whom other clinical factors cannot be used for risk stratification (e.g., young obese patients). Classic risk factors may exercise greater influence over disease extent and clinical presentation.

One theory that might justify our findings is based on the fact that APN only becomes present on the vessel wall after some vascular injury has been sustained. This would promote migration of APN to the subendothelial layer, where it would exert its antiatherogenic and anti-inflammatory effects by stimulating nitric oxide production, inhibiting TNF-α secretion, and blocking expression of endothelial adhesion molecules, thus preventing neointimal formation (21,22). Circulating APN levels correlate inversely with levels of C-reactive protein (12), an acute phase marker of vascular inflammation that inhibits nitric oxide production and thus perpetuates the inflammatory process, a phenomenon closely related to the development of ACS (23,24). Studies have shown that, during the earliest stages of ACS, a marked reduction in serum APN levels occurs for up to 72 h, with near-complete recovery within 7 days. The precise underlying mechanism has yet to be determined, but atherosclerotic plaque rupture is believed to lead to excessive consumption of APN in an attempt to resolve the vascular inflammatory process (25).

Regarding the extent of CAD, studies in diverse populations have correlated serum levels of APN and its isoforms with disease extent estimated by different angiographic scores. Von Eynatten et al. (26,27) in a comparison between a control group of individuals without CAD and a group of patients with single-vessel, two-vessel, or three-vessel disease, demonstrated a correlation between extent of CAD and HMW APN and APN levels. Inoue et al. (10) found a similar association, namely, a statistically significant difference in HMW APN levels between patients with single-vessel and those with multi-vessel disease; however, their sample did not include diabetes patients or patients with acute coronary syndrome. More recently, Azizi Ghanbari et al. (28) evaluated patients undergoing cardiac catheterization (again, excluding diabetes patients) and found a gradual reduction in APN values with increasing number of affected vessels, particularly in men.

In our study, we also used the Duke Jeopardy Score to evaluate extent of CAD. This score correlates well both with other angiographic scoring systems and with plaque area and plaque burden as measured by intracoronary ultrasound (29). It is also easy to use, has good reproducibility, and is able to estimate myocardial area at risk and, consequently, extent of CAD.

Several studies have compared APN with disease extent as quantified by anatomic scores (20,26,27,30,31); most demonstrated some correlation with serum APN levels. The most robust study (20), which included 399 patients and used the Gensini score, found no correlation between these variables. However, it bears stressing that we used a different angiographic score as well as categorized scores into three subgroups (low, intermediate, and high). Most patients in our sample were categorized into the low-score subgroup (n=138), while very few were in the high-score subgroup (n=28). Therefore, although we did not detect a significant difference between the groups, we cannot rule out the possibility of such a difference having gone undetected due to an insufficient sample size.

The current literature on this topic remains conflicting and certainly highlights the need for additional, large-scale studies. Within this context, our series of over 400 cases contributes by sustaining consistent association between lower serum levels of APN and HMW APM within this population with rigorously defined coronary artery disease. In conclusion, serum levels of APN and HMW APN were not different according to clinical presentation or extent of CAD, as estimated by number of affected vessels and Duke Jeopardy Score. Nevertheless, levels of both markers were significantly reduced in the presence of obstructive CAD in this group of patients with high cardiovascular risk, as demonstrated previously.
